# Intracellular pH Regulates TRAIL-Induced Apoptosis and Necroptosis in Endothelial Cells

**DOI:** 10.1155/2017/1503960

**Published:** 2017-08-13

**Authors:** Zhu-Xu Zhang, Ingrid Gan, Alexander Pavlosky, Xuyan Huang, Benjamin Fuhrmann, Anthony M. Jevnikar

**Affiliations:** ^1^Matthew Mailing Centre for Translational Transplantation Studies, London Health Sciences Centre, London, ON, Canada; ^2^Multi-Organ Transplant Program, London Health Sciences Centre, London, ON, Canada; ^3^Division of Nephrology, Department of Medicine, Western University, London, ON, Canada; ^4^Department of Pathology, Western University, London, ON, Canada; ^5^Department of Microbiology & Immunology, Western University, London, ON, Canada

## Abstract

During ischemia or inflammation of organs, intracellular pH can decrease if acid production exceeds buffering capacity. Thus, the microenvironment can expose parenchymal cells to a reduced extracellular pH which can alter pH-dependent intracellular functions. We have previously shown that while silencing caspase-8 in an *in vivo* ischemia reperfusion injury (IRI) model results in improved organ function and survival, removal of caspase-8 function in a donor organ can paradoxically result in enhanced receptor-interacting protein kinase 1/3- (RIPK1/3-) regulated necroptosis and accelerated graft loss following transplantation. In our current study, TRAIL- (TNF-related apoptosis-inducing ligand-) induced cell death *in vitro* at neutral pH and caspase-8 inhibition-enhanced RIPK1-dependent necroptotic death were confirmed. In contrast, both caspase-8 inhibition and RIPK1 inhibition attenuated cell death at a cell pH of 6.7. Cell death was attenuated with mixed lineage kinase domain-like (MLKL) silencing, indicating that MLKL membrane rupture, a distinctive feature of necroptosis, occurs regardless of pH. In summary, there is a distinct regulatory control of apoptosis and necroptosis in endothelial cells at different intracellular pH. These results highlight the complexity of modulating cell death and therapeutic strategies that may need to consider different consequences on cell death dependent on the model.

## 1. Introduction

Inflammatory stress can mediate various forms of cell death, which are relevant to diverse forms of human disease. Cell death is particularly relevant to organ transplantation as stress includes both temporary hypoxia as the organ is retrieved and inflammation associated with reperfusion following reestablishment of blood flow [1, 2]. Apoptosis relies on an intracellular cascade of caspase family members which leads to the formation of membrane-bound apoptotic bodies that are eliminated by noninflammatory phagocytosis such as kidney injury molecule-1- (KIM-1-) mediated cell clearance [3, 4]. Recently, regulated forms of necrosis have been described. Regulated necrosis results in cell lysis and intense inflammation in response to the release of cell contents. The scope of regulated necrosis has evolved rapidly to include not only necroptosis but also ferroptosis, oxytosis, parthanatos, and pyroptosis and others [5].

Necroptosis is dependent on receptor-interacting protein kinase 1/3 (RIPK1/3) to mediate cell death [6, 7]. This pathway is induced by various ligands including TNF*α*, FasL, and Toll-like receptor (TLR) engagement. Of note, TNF-related apoptosis-inducing ligand- (TRAIL-) mediated apoptosis has long been described as a method to induce cancer cell death through the activation of caspase-8 [8, 9]. More recently, TRAIL has been shown to also induce necroptosis in cancer cells [10–12]. Interestingly, cells can be sensitized to necroptotic death through inhibition or alteration of endogenous proteins such as TNF receptor-associated factor 2 (TRAF2) [13] or cellular inhibitor of apoptosis 1/2 (CIAP1/2) [14]. Additionally, there are interactions between the components of apoptosis and necroptosis pathways. Necroptosis can be spontaneously induced through genetic deletion of caspase-8 [15–17] which is embryonically lethal, as well as through elimination of Fas-associated death domain protein (FADD) [18], or by intracellular oligomerization of RIPK3 [19]. Caspase-8 regulates necroptosis primarily through cleavage and inactivation of the necroptosis-inducing molecules RIPK1 and RIPK3 [16]. RIPK3 mediates activation of mixed lineage kinase domain-like (MLKL) [20, 21], the effector molecule that ultimately induces necroptotic death by inducing membrane breakdown [22].

Necroptosis has been implicated in a variety of inflammatory diseases which have been reviewed [23–25]. Of interest, inhibition of necroptosis has been shown to be beneficial in cardiac [26] and renal ischemia reperfusion injury (IRI) [27]. In addition to others, we have shown that silencing caspase-8 by siRNA in the kidneys can improve function and prolong survival in renal IRI models [28, 29]. We have also demonstrated that elimination of RIPK3 in donor organs is beneficial following renal [30] or cardiac [31] transplantation by preventing necroptosis. However, silencing of caspase-8 was not of a benefit in renal transplantation and increased inflammatory injury associated with increased necroptosis [30]. Collectively, these results suggested that IRI and transplantation did not represent identical models in terms of caspase-8 control. It has been described that TRAIL-induced necrotic cell death can occur without caspase-8 inhibition in low extracellular pH [11], which was a RIPK1, RIPK3, and poly(ADP-ribose) polymerase 1- (PARP-1-) dependent form of cell death [12, 32]. Parenchymal cells are exposed to acidic pH in pathological conditions in the brain, kidney, and heart [33, 34]. pH in organ quickly falls below 7 after ischemia [35–39]. pH changes in cells might thus account for our observations of the somewhat paradoxical benefit of caspase-8 inhibition in acute ischemic models and the clear lack of benefit in a more chronic model, in which acute pH changes have likely resolved. Indeed, the introduction of pulsatile perfusion of buffer solutions to clinical organ preservation strategies have provided a benefit by minimizing intraorgan pH changes and tissue injury [40].

In the present study, we show that inhibition of caspase-8 promotes TRAIL-mediated necroptosis at a normal physiological extracellular and intracellular pH, but not at an acidic pH in murine endothelial cells. Our findings also show that regulated death at an acidic pH relies not only on the function of RIPK1, caspase-8, but also PARP-1, implicating parthanatos [41] as well as apoptosis and necroptosis. These findings provide important new insight into IRI in which caspase-8 inhibition exerts a protective role in a low pH microenvironment, but the same strategy can become proinflammatory as pH normalizes.

## 2. Materials and Methods

### 2.1. Microvascular Endothelial Cell (MVEC) Culture

MVECs from mouse hearts were isolated and developed as previously described [31]. MVEC phenotype was confirmed by staining with anti-CD31, anti-CD102, and anti-CD105 (eBioscience) [31]. Cells were grown in complete EGM-2 MV containing 5% FBS, 0.04% hydrocortisone, 0.4% hFGF-b, 0.1% VEGF, 0.1% R3-IFG-1, 0.1% ascorbic acid, 0.1% hEGF, and 0.1% GA-1000 (Lonza).

### 2.2. pH Conditions

EBM-2 media without growth factors (Lonza) with 50 mM HEPES (Wisent) was adjusted to either pH 7.4 or 6-6.7 using HCl. Cells were grown to monolayers and incubated in this media with the indicated pH. Intracellular pH change was detected using pHrodo red pH indicator (ThermoFisher) and monitored using IncuCyte live-cell imager (Essen Bioscience). High fluorescence intensity is indicative of a lower intracellular pH.

### 2.3. Western Blot

Protein was isolated from heart tissue using whole cell lysis buffer (20 mM HEPES, 0.4 mM NaCl, 1 mM EDTA, 1 mM EGTA, 1 mM DTT, and 1 mM PMSF). Protein concentration was determined using Bio-Rad protein assay (Bio-Rad). Sample buffer (2ME, glycerol, bromophenol blue, and Tris-HCl) was added to the protein and was separated by gel electrophoresis. Protein was transferred to a nitrocellulose membrane using the iBlot dry transfer system (Invitrogen). Membranes were incubated with rabbit anti-RIPK1 (EPR19697, Abcam), polyclonal rat anti-mouse MLKL (Milipore), rabbit anti-Glyceraldehyde 3-phosphate dehydrogenase (GAPDH, Proteintech Group), or anti-*β*-actin (Sigma Aldrich). Protein was visualized using secondary anti-IgG with conjugated horseradish peroxidase and chemiluminescent substrate (Millipore).

### 2.4. Small Interference RNA (siRNA)

MVECs were transfected with MLKL siRNA or scrambled (nonsense) siRNA (Santa Cruz Biotech, CA) with Lipofectamine 2000 (Invitrogen, Carlsbad, CA). Cells were transfected with 2 *μ*g of the siRNA in serum-reduced medium for 5 hours and then incubated in complete medium for 24 hours per manufactory protocols. Cells were prepared for subsequent analysis and experiment.

### 2.5. Real-Time PCR

Total RNA was extracted from tissue or cells by Trizol extraction (Invitrogen). cDNA was generated from RNA using Superscript II (Invitrogen). Primers used for real-time PCR include the following: MLKL 5′-TTG CTG GGA GCA AAT AGC-3′ and 5′-GAG TTT GAG CCA GCC TGT-3′ and *β*-actin 5′-CCA GCC TTC CTT CCT GGG TA and 3′-CTA GAA GCA TTT GCG GTG CA. Real-time quantitative PCR was performed on standardized quantities of cDNA using the SYBR QPCR mixture. *β*-Actin amplification was used as the endogenous control. The normalized delta threshold cycle value and relative expression levels (2^−∆∆Ct^) were calculated per the manufacturer's protocol.

### 2.6. Cell Death Assay

MVECs were grown to a monolayer in a 96-well plate (2 × 10^4^ cells/well) and treated with 100 ng/ml of recombinant mouse TRAIL (Peprotech), 100 nM second mitochondria-derived activator of caspase (SMAC) mimetic compound (SMC, GDC-0152, Selleckchem), 50 *μ*M zIETD-fmk, 20 *μ*M Necrostatin-1s (Nec-1s), and 50 *μ*M PARP-1 inhibitor 3-aminobenamide (3-ABA, Calbiochem). At the time of treatment, 100 nM of the DNA-intercalating molecule, Sytox green (Invitrogen), was added to detect cell death. Sytox fluorescence (positive cells/well) was measured every hour using IncuCyte live-cell imager (Essen Bioscience).

### 2.7. Statistical Analysis

Data was compared using Student's *t*-test for unpaired values. Data was presented as mean ± standard deviation (SD). *p* values below 0.05 were considered to be significantly different.

## 3. Results

### 3.1. Intracellular pH Was Decreased in MVEC Grown under Acidic Conditions

**Figure 1:**
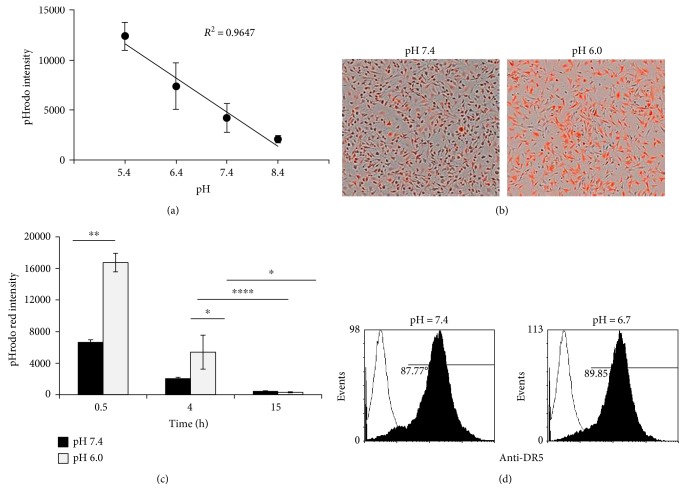
MVECs express high levels of DR5 and respond to extracellular pH changes. (a) MVECs in triplicates in a 96-well plate were stained with the pH sensitive dye pHrodo red (ThermoFisher) for 30 minutes before being incubated in the medium at pH 5.4, 6.4, 7.4, and 8.4 for 30 minutes. The pHrodo red fluorescence intensity in each well was quantified by IncuCyte live-cell imager. Higher fluorescence intensity is indicative of a lower intracellular pH and appears red. (b, c) Time course of pHrodo red fluorescence intensity. MVECs in triplicates were stained with pHrodo red and incubated in the medium at pH 6 or 7.4 for different time. pHrodo red fluorescence intensity was monitored by IncuCyte live-cell imager. Image (20x) and quantification result represented one of four experiments, and similar results have repeated four times. ^∗^*p* ≤ 0.05, ^∗∗^*p* ≤ 0.01, and ^∗∗∗∗^*p* ≤ 0.0001 (*t*-test). (d) MVEC expression of the TRAIL receptor DR5 at pH 7.4 and pH 6.7. Expression of DR5 was detected by anti-DR5-PE and analyzed by flow cytometry. Histogram shown is representative of three experiments.

MVECs were grown to monolayers, and intracellular pH changes in pH 5.4–8.4 medium were detected by pHrodo red fluorescence indicator (Figure 1(a)). Increased fluorescence intensity in cells at acidic pH demonstrated that MVEC intracellular pH was directly related to the pH of the environment (Figures 1(b) and 1(c)). However, intracellular pH restored towards neutral pH following time as indicated by decreased fluorescence intensity in cells (Figure 1(c)). MVEC expressed a high level of TRAIL receptor DR5, but this did not change under acidic conditions (Figure 1(d)).

### 3.2. Caspase Inhibition Did Not Induce Necroptosis in MVEC under Acidic Conditions

**Figure 2:**
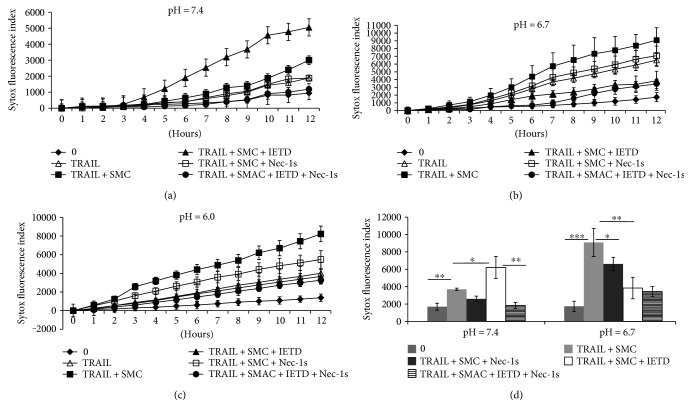
MVEC cell death modality is pH dependent. (a) MVECs (triplicates) were treated with 100 ng/ml TRAIL, 100 nM SMC, 50 *μ*M zIETD-fmk, and 20 *μ*M Nec-1s at pH 7.4. The kinetic cell death response of MVEC to TRAIL is measured by Sytox green and IncuCyte live-cell imager. (b) The kinetic cell death response of MVEC to TRAIL at pH 6.7. MVECs were treated with 100 ng/ml TRAIL, 100 nM SMC, 50 *μ*M zIETD-fmk, and 20 *μ*M Nec-1s at pH 6.7. (c) The kinetic cell death at pH 6.0. (d) Conclusion of cell death at 12 hours. Data shown as mean of triplicates ± standard deviation (SD) of fluorescence intensity of Sytox. Similar results were obtained in nine repeated experiments. ^∗^*p* ≤ 0.05, ^∗∗^*p* ≤ 0.01, and ^∗∗∗^*p* ≤ 0.001 (*t*-test).

To test if the microenvironment pH could affect the modality of MVEC death, necroptosis was induced by a combination of SMAC mimetic compound (SMC), TRAIL, and caspase-8 inhibitor IETD-fmk. The RIPK1 inhibitor Nec-1s, which blocks necroptosis, was added to cultures at pH 7.4 (Figures 2(a) and 2(d)), pH 6.7 or pH 6.0 (Figures 2(b), 2(c), and 2(d)). At a normal pH of 7.4, TRAIL plus SMC induced a low level of cell death and predictably underwent necroptosis with caspase-8 inhibition using IETD-fmk-enhanced TRAIL-mediated cell death (with IETD 6209 ± 1274 versus without IETD 3701 ± 127 Sytox-positive cells at 12 hours, *p* = 0.013). TRAIL/IETD-induced MVEC death could be maximally inhibited by the addition of Nec-1s (1846 ± 340, *p* = 0.002), confirming that this was RIPK-mediated necroptosis. The large reduction of cell death using Nec-1s in TRAIL/SMC cells suggests that the primary form of death is necroptosis, although the residual amount of cell death might be attributed to apoptosis or other forms of cell death. MVEC at pH 6.7 underwent substantial cell death following TRAIL plus SMC treatment alone (untreated 1736 ± 592 versus 9088 ± 1609 Sytox-positive cells at 12 hours, *p* = 0.0005). However, in marked contrast to results at pH 7.4, addition of the caspase-8 inhibitor IETD-fmk did not increase death but substantially blocked cell death (3842 ± 1236 Sytox-positive cells, *p* = 0.004). As well, there was a minimal effect with Nec-1s alone in TRAIL/SMC cells. Cell death at pH 6.0 (Figure 2(c)) is similar to the result at pH 6.7. This data suggests that TRAIL engagement is able to induce cell death at normal and acidic pH environment but that low pH skews cell death to apoptosis. Furthermore, in distinct contrast to pH 7.4, MVEC death can be blocked by caspase-8 inhibition while attempting to attenuate MVEC death at pH 7.4 by caspase-8 inhibition resulted in more MVEC death through necroptosis.

### 3.3. TRAIL-Induced Cell Death at Acidic Condition Is Dependent on PARP-1

**Figure 3:**
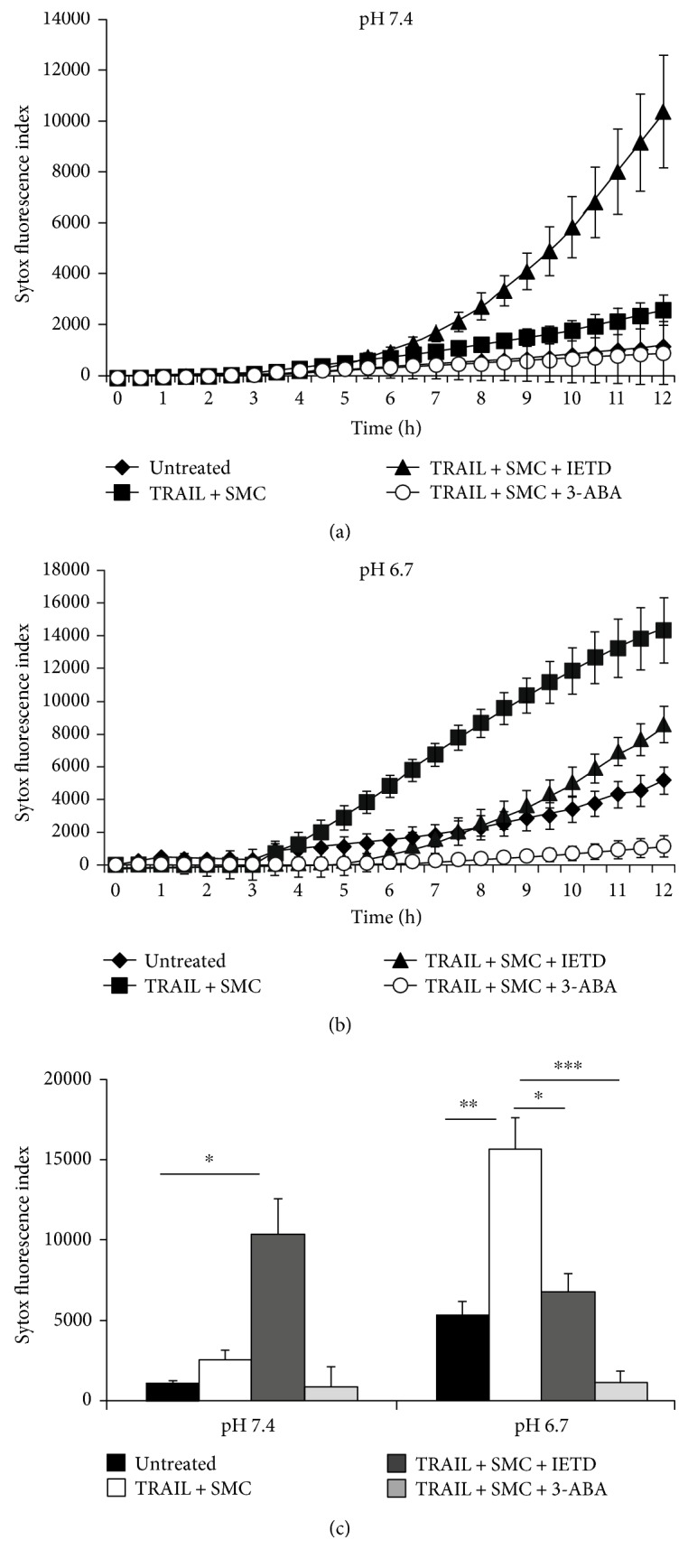
MVEC death at acidic condition is dependent on PARP-1. (a) MVECs (triplicates) were treated with 100 ng/ml TRAIL, 100 nM SMC, 50 *μ*M zIETD-fmk, and/or 3-ABA at pH 7.4. Kinetic cell death responses were measured by Sytox green staining and quantified by IncuCyte live-cell imager. (b) MVECs were treated with TRAIL, SMC, zIETD-fmk, and/or 3-ABA at pH 6.7. (c) Conclusion of cell death assay at 12 hours. Data shown as mean of triplicates ± SD of fluorescence intensity of Sytox. Similar results were obtained in three repeated experiments. ^∗^*p* ≤ 0.05, ^∗∗^*p* ≤ 0.01, and ^∗∗∗^*p* ≤ 0.001 (*t*-test).

As noted by others [32], necrosis in acid conditions appears to be dependent on PARP-1 activation in cancer cells. To test this in MVEC, cells were treated with the caspase-8-specific inhibitor zIETD-fmk along with the PARP-1 inhibitor 3-ABA and exposed to TRAIL at pH 7.4 (Figures 3(a) and 3(c)) and pH 6.7 (Figures 3(b) and 3(c)). At pH 7.4, MVEC underwent necroptosis following the addition of zIETD-fmk (TRAIL/IETD 10368 ± 2208 versus untreated 1136 ± 136 Sytox-positive cells, *p* = 0.014). The addition of TRAIL/SMC alone increased death minimally by 12 hours, although the PARP-1 inhibitor 3-ABA reduced death below baseline. In contrast, as noted previously at pH 6.7, TRAIL/SMC-induced death could be partially recovered by both zIETD (without IETD 14328 ± 1990 versus with IETD 8581 ± 1100, *p* = 0.012). However, the addition of 3-ABA (1146 ± 672, *p* = 0.0006) reduced cell death to baseline, indicating that PARP-1-dependent cell death as well as apoptosis was occurring under acidic conditions.

### 3.4. RIPK1 Cleavage under pH 7.4 and 6.7

**Figure 4:**
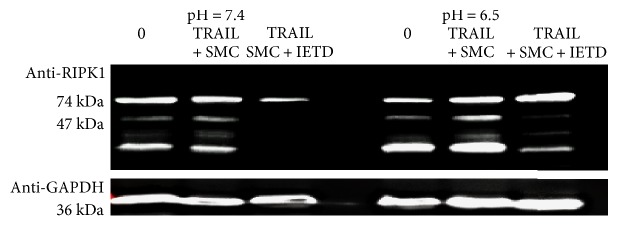
RIPK1 cleavage at pH 7.4 and 6.7. Equal numbers of MVEC were induced to cell death as described in Figure 2 at pH 7.4 and 6.7. MVECs were harvested 8 hours after and analyzed for RIPK1 cleavage (74 kDa and 47 kDa) by anti-RIPK1 and Western blot. Anti-GAPDH was used as loading control. The experiment for cell death and Western blot has been repeated three times and similar result was obtained.

A previous study has shown that RIPK1 is not cleaved under acidic pH conditions in HT29 cells, which may explain why RIPK1-dependent necrosis can occur at acidic conditions [12]. We next determined if TRAIL treatment under both physiologic and acidic conditions results in RIPK1 cleavage. Interestingly, RIPK1 was cleaved on TRAIL treatment at pH 7.4 as well as pH 6.7 (Figure 4). The cleavage of RIPK1 remained caspase-8 dependent as caspase-8 inhibition by z-IETD-fmk prevented the RIPK1 cleavage under normal and acidic conditions (Figure 4).

### 3.5. TRAIL-Induced Cell Death at Acidic Condition Is MLKL Dependent

**Figure 5:**
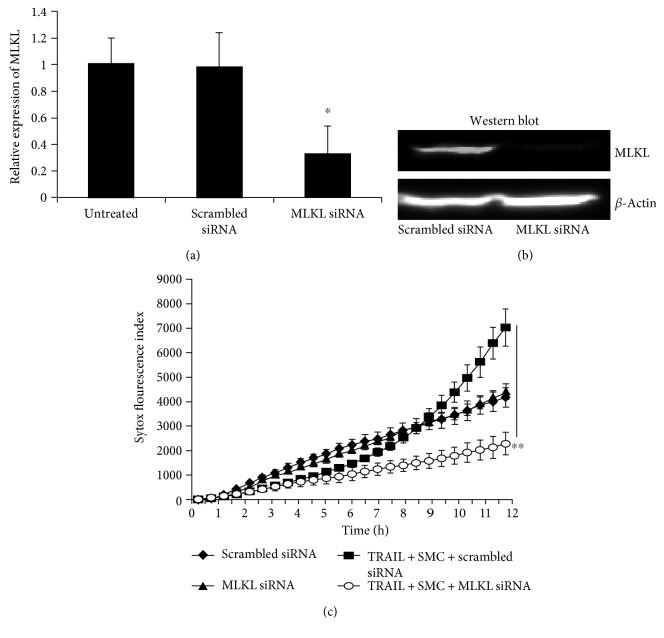
Acidosis-regulated necroptosis is dependent on MLKL. (a) MLKL siRNA treatment decreases MLKL mRNA expression as confirmed by real-time PCR analysis as described in Materials and Methods (*n* = 3). (b) Decrease of MLKL protein was analyzed by anti-MLKL in Western blot. (c) MLKL siRNA silence attenuates cell death at pH 6.7. siRNA-treated MVECs (triplicates) were induced death with TRAIL and SMC at pH 6.7. Kinetic cell death responses were quantified by Sytox green and IncuCyte live-cell imager. Similar results were obtained in three repeated experiments. ^∗^*p* ≤ 0.05 and ^∗∗^*p* ≤ 0.01 (*t*-test).

MLKL is the terminal effector molecule for necroptosis as it induces cell membrane rupture after phosphorylation by RIPK3 [20, 42]. To test the contribution of this executioner protein in TRAIL-induced necroptosis under acidic conditions, MLKL was silenced in MVEC using siRNA as confirmed by PCR and Western blot analyses (Figures 5(a) and 5(b)). As shown in Figure 5(c), TRAIL-induced cell death was attenuated in MLKL siRNA-treated cells at pH 6.7 (Sytox-positive cells at 12 hours: 2277 ± 456 versus 7033 ± 753 in scrambled siRNA-treated cells, *p* = 0.002), confirming that MLKL-dependent necroptosis occurs under acidic conditions.

## 4. Discussion

Necroptosis contributes to the pathogenesis of many inflammatory diseases. We have previously shown that RIPK3-dependent necroptosis results in increased inflammation and reduced survival in renal and heart transplants. This reduced survival was tightly associated with greater organ injury and release of proinflammatory cell damage-associated molecular patterns (CDAMPs) [30, 31]. We had previously noted that inhibition of caspase-8 yields a benefit during IRI. Caspase-8 silencing in a renal IRI model provided injury protection and improved short-term survival [28, 29]. As organ injury has been shown to improve by targeting apoptosis cell death [28, 29, 43–46], we noted that caspase-8 silencing by siRNA in a kidney allograft model did not have an expected benefit and indeed resulted in massive *in vivo* necrosis and accelerated graft rejection [30]. These disparate findings using the same intervention in two different models may allude to many differences between acute (IRI) and chronic (transplant) models, clearly diverge in response to selective caspase-8 targeting. Parenchymal cells deprived of oxygen and nutrients in acute IRI and in the early phase of transplant may respond similarly to hypoxia, but IRI resolves quickly while alloimmunity persists in transplantation. These models may highlight the importance of the cellular microenvironment on cell death.

Ischemic cells undergoing anaerobic metabolism generate lactic acid and experience a subsequent drop in intracellular pH. Our study has clearly demonstrated that changes in the pH of the microenvironment of endothelial cells lead to intracellular pH change and altered the function of caspase-8 and other proteins. The mechanism balancing cell death and in particular, apoptosis and necroptosis in endothelial cells changes under acidic conditions. Apoptosis and necroptosis occur simultaneously in response to TRAIL activation in endothelial cell at acidic condition. Our findings provide an important new insight into our observation that caspase-8 inhibition can play a protective role during IRI related to a low intracellular pH and microenvironment, while paradoxically becoming proinflammatory within transplantation in a normal pH environment.

Death in endothelial cells at an acidic pH relies on the function of RIPK1 and caspase-8 (Figures 2 and 3). PARP-1 also appears to be affected by the cellular microenvironment, playing a limited role in necroptosis at pH 7.4 [47, 48], and evident in the low pH cell death observed here (Figure 3) and others [32]. Given the distinct function of caspase-8 and influence of PARP-1, the precise nature of cell death we have observed at low pH which we have termed “acidonecrosis” does not fit well as classical necroptosis or apoptosis. A previous study showed that TRAIL mediates apoptosis through activation of caspase, cytochrome C release, and PARP-1 cleavage in human tumor cells at acidic pH [49, 50]. Other studies have shown that TRAIL can easily induce cell death in tumor cells at pH 6.5 but to a significantly lesser extent at pH 7.4 [11, 12, 32, 49]. In contrast, our study shows that MVEC readily underwent classical necroptosis at pH 7.4 as well as “acidonecrosis” at pH 6–6.7 after TRAIL treatment (Figure 2). Differences in certain death modalities between cell types might allow organ-specific targeted therapies to be more effective.

The mechanism of “acidonecrosis” remains unclear. RIPK1, RIPK3, caspase-8, and PARP-1 all appear to be necessary to induce necrotic death with TRAIL at low pH. Intriguingly, the question whether PARP-1 is activated upstream or downstream of RIPK1 remains uncertain. RIPK1 activation has previously been shown to be the upstream of PARP-1 activation following DNA alkylation [51]. However, inhibition of RIPK1 is also able to inhibit PARP-1 activation and apoptosis-inducing factor (AIF) release from mitochondria following *β*-lapachone-mediated regulated necrosis [52]. A unique and novel finding in the present study is the inhibition of TRAIL-mediated cell death following the silencing of MLKL by siRNA in low pH conditions (Figure 5). MLKL activation occurs downstream of RIPK1/RIPK3 phosphorylation and is both necessary and sufficient to induce necroptosis [21, 22, 42, 53]. An important question remains: if MLKL activation is sufficient to induce necroptosis normally, why is PARP-1 activation required for “acidonecrosis”? A recent study may provide some insight in this question, as necroptosis within a transplanted kidney graft can remarkably cause distant lung parthanatos, also by an unknown mechanism [41]. This important observation may suggest that the acidonecrosis we observed *in vitro* is TRAIL-mediated necroptosis that leads to a paracrine form of parthanatos. In addition, a previous study showed TRAIL-mediated apoptosis through PARP-1 cleavage at low pH [49, 50]. Detailed studies would be required to detail potential signals involved, namely, whether this occurs by membrane-bound vesicles capable of fusing with other cells or if cytoplasmic contents can induce this. Clearly, further studies are required to determine the essential roles of caspase-8, PARP-1 activation, and MLKL during “acidonecrosis.” However, it remains controversial whether PARP-1-mediated necrosis/parthanatos involves RIPK1/3 and depends on specific cell types used in different studies [32, 47, 51]. Our data suggested that PARP-1 participates in “acidonecrosis” (Figure 3) in MVEC. It is possible that PARP-1 may be the downstream effect of both apoptosis and necrosis at acidic pH [32, 54].

Under conditions of acidic intracellular pH, cellular functions such as ion transport, enzyme activities, protein synthesis, and DNA synthesis can be diminished or altered [33, 34, 55, 56]. Acidic pH conditions may result in a decreased intracellular level of K^+^ because of H^+^/K^+^ pump activity. A decrease of K^+^ concentration results in the activation of caspases and nucleases and thus leads to DNA damage [57, 58] and apoptosis [59–61]. This mechanism might explain why acidosis induces apoptotic cell death in various types of cells, including endothelial cells by activating caspases [50, 62–64]. However, other studies showed that acidosis protects endothelial cells from apoptosis by blocking caspase activation or enhancing expression of antiapoptotic molecules [65–68]. It requires further study to define different types of cell death program in different cell types.

A recent study has shown that acidosis-induced necroptosis in neurons is dependent on acid-sensing ion channel 1a- (ASIC1a-) mediated RIPK1 phosphorylation [69]. Acid-sensing ASIC1a mediation of RIPK activation may thus explain how RIPKs are activated even in the presence of caspase-8 during acidosis. A previous study also showed that RIPK1 is cleaved at normal pH but not cleaved at acidic pH in tumor cells [12]. While these results might explain why RIPK1 can induce necroptosis despite in the presence of caspase-8 activity [11, 12], our data showed that RIPK1 is partially cleaved at normal and acidic pH conditions (Figure 4). This indicates caspase-8-mediated RIPK1 cleavage at pH 7.4 is not altered when pH drops to acidic condition. It is likely that TRAIL induces apoptosis and necroptosis simultaneously at acidic pH in MVEC, as RIPK1 is only partially cleaved (Figure 4) and the inhibition of either caspase-8 or RIPK1 could attenuate acidonecrosis (Figures 2 and 3).

## 5. Conclusions

This study highlights the importance of the cellular microenvironment on the magnitude, progression, and outcome of several forms of programmed cell death. For cancer therapeutics, the cell type and surrounding tumor microenvironment appear to play important roles in increasing the sensitivity of cells to different modalities of cell death, which is of central importance in maximizing cancer cell death. In contrast, with the goal in transplantation being the opposite with maximum reduction of cell death, the nature and timing of antideath strategies during organ procurement and transplantation becomes complex as we test normothermic and hypothermic perfusion strategies as well as different perfusion solutions with altered buffering capacity to minimize ischemic injury. The extracellular microenvironment including pH clearly regulates the intracellular and subsequently the cell death programs that result in inflammation. Blocking multiple pathways of cell death including apoptosis and necroptosis and others perhaps may be more effective in preventing IRI-induced cell death and organ injury during transplantation than targeting a single-cell death pathway.
